# Metropolitan Hospital Dinner

**Published:** 1894-05-05

**Authors:** 


### METROPOLITAN HOSPITAL DINNER.

Few appeals made in connection with recent festival dinners of London hospitals have been characterised by greater urgency than that of the Metropolitan, which has just been put forward in connection with last Monday's annual gathering at the Hotel Metropole. Since the opening of the institution in 1887, in the midst of the poor and thickly-populated districts of Shoreditch, Hackney, Haggerston, Bethnal Green, Hoxton, Dalston, and De Beauvoir Town, its record has been particularly disappointing. Its financial position has always been unsatisfactory, and, indeed, there seems some prospect that it may even yet become worse. The Metropolitan Hospital has always been regarded as the special *protégé* of the City. It is so historically, as it was formerly situate in Devonshire Square, and since its removal in 1887 to a site where it is, or rather might be of far greater use, the City has been well represented in the subscription list. Only last year the Corporation voted £105, and some eight or nine companies also contributed, the Goldsmiths' heading the list with £200. But these large sums are quite exceptional, and indeed as the annual subscription list only amounts to £600 a-year, when really about £2,000 is needed to make the hospital finance really satisfactory, and as, moreover, the small endowment of the institution is less than £300, it is impossible to carry on the work without material aid from the wealthy. The hospital is, of course, one of the most recent in point of construction, and is excellently built, but only 54 beds out of a possible 160 are open, and, indeed, never as yet have more than 78 been available, and 24 were closed in consequence of pecuniary straits last year. Thus considerably more than half the wards are unfurnished, and, in consequence, if ever the existing building is to be utilised to its fullest extent, a very large initial expenditure would have to be incurred. But this is not all that could be done in the way of extension of work, provided the funds existed to carry it into effect. The hospital stands on its own freehold property of two and a-half acres in the Kingsland Road, Shoreditch, and is capable of being very materially enlarged so as to afford accommodation for about 400 patients besides special provision for the nurses. At present nothing can be done, for the hospital is heavily in debt. There is a mortgage of £10,000 on the building, and no less than £3,594 8s. is owing to tradesmen, and to clear off the latter charge the present appeal is being made. As to the need for the hospital in its present position, there is no, and never has been any, question. The institution is in a thoroughly poor neighbourhood, and from the nature of its situation does not prominently come under the notice of the charitable public. Yet it does a good work, though sadly hampered. Even last year, when the available number of beds had diminished, 857 as against 894 in-patients in 1892-3, were treated; while the out-patients' total rose from 71,754 to 73,283. For the most part these latter are very poor, as a reference to the hospital books shows. The institution is free, and it is worthy of note that the in-patients have here all their groceries provided for them without charge, an eminently desirable proceeding in so poor a neighbourhood, but one which has much contributed to the size of the debt to tradesmen. At present it is impossible for the hospital authorities to much longer avoid a thorough renovation of the interior surfaces of the hospital, but they are naturally desirous of deferring the day as long as possible, as they are ever now in financial straits. The dinner at the Hotel Metropole was presided over by the Lord Mayor, who has taken a warm interest in the present appeal, and has fully realised the serious position in which the hospital at present stands. Amongst the company were Mr. Alderman and Sheriff Dimsdale, Mr. Joseph Fry, the Rev. R. H. Hadden, Mr. T. Berens, Mr. J. R. Pike, the Rev. R. S. Hassard, Mr. Coleman Defries, Baron de Bush, Mr. S. H. Flindt, Mr. T. Hartley, Mr. J. Westley, Mr. C. J. Thomas, Mr. J. B. Ingle, Dr. Fotherby, Dr. H. H. Tooth, Mr. D. H. Goodsall, Mr. J. Hewitt, Major Wood, Mr. H. Lovegrove, Mr. R. H. Cunningham, Mr. W. Goodsall, Mr. Mason, Mr. Starling, Mr. Warner, Mr. Littlehales, and Mr. C. H. Byers (Secretary). In proposing the toast of the evening, "Prosperity to the Hospital," the Chairman said he knew of no institution of the kind which had greater claims upon the citizens of London and upon the public at large than the Metropolitan Hospital. Originally in Devonshire Square in the City, it had been transferred to the centre of a neighbourhood which was inhabited by about 250,000 of the poorer classes, whose homes were entirely unsuited for the proper treatment of illness. They had land enough for a hospital with 400 beds, but to complete its erection would require a great deal of money. They were very short of funds; they knew, indeed, scarcely which way to turn, and unless some help was given he knew not what would be the result. He commended the hospital to their warm support, but he feared that the day was not far distant when the Lord Mayor might have to give up the presidency. ("No.") Well, he should be glad if they would make it unnecessary for any Lord Mayor to do so. Mr. Joseph Fry, the Chairman of the Board, said that unless more money came in he did not know what they would do. It might be that they would have to close the hospital altogether, a proposal, he hoped, they would not be compelled to entertain. Other toasts followed, and the Secretary then announced subscriptions to the amount of £2,209, including £500 from Baron de Hirsch, and it was also stated that Mr. H. Spicer having left £11,500 to be set aside specially for the benefit of convalescent patients, Mr. Passmore Edwards, in furtherance of this project, had promised to build for the hospital a Convalescent Home in a suitable place.

